# Supplementary material for: Vocational Identity Status in Chinese Emerging Adults with and without Hearing Impairment: Latent Profiles and Relationships with Self-Esteem and Subjective Well-Being
Source: Int J Environ Res Public Health. 2022 Nov 4;19(21):14473. doi: 10.3390/ijerph192114473 (PMC9654180; doi:10.3390/ijerph192114473)
Supplement: Supplementary file 1 [file ijerph-19-14473-s001.zip › ijerph-1952510-supplementary.pdf]

Table S1. Invariance results for aligned factor loadings and intercepts parameters (numbers in parentheses show significant non-invariance for the parameter)

Parameter	Factor loadings	Intercepts
CCM1	0 1	0 1
CCM2	0 1	0 1
CCM3	0 1	0 1
CCM4	0 1	0 1
ICC5	0 1	（0）（1）
ICC6	0 1	0 1
ICC7	0 1	0 1
ICC8	0 1	0 1
ICC9	0 1	0 1
ICC10	0 1	（0）（1）
CF11	0 1	0 1
CF12	0 1	0 1
CF13	0 1	0 1
CF14	0 1	0 1
CSd16	0 1	0 1
CSd17	0 1	0 1
CSd18	0 1	0 1
CSd19	0 1	0 1
CSd20	0 1	0 1
BCE21	0 1	0 1
BCE22	0 1	0 1
BCE23	0 1	0 1
BCE24	0 1	（0）（1）
BCE25	0 1	0 1
DCE15	0 1	0 1
DCE26	0 1	0 1
DCE27	0 1	0 1

DCE28	0 1	0 1
DCE29	0 1	0 1
DCE30	0 1	0 1

Note: 0 refers to the hearing group, 1 refers to the hearing impairment group
